# Collaboration between general practitioners and mental health care professionals: a qualitative study

**DOI:** 10.1186/1752-4458-5-13

**Published:** 2011-05-23

**Authors:** Terje Fredheim, Lars J Danbolt, Ole R Haavet, Kari Kjønsberg, Lars Lien

**Affiliations:** 1 Centre for Psychology of Religion, Innlandet Hospital Trust (SIHF), Hamar, Norway; 2 Department of General Practice, University of Oslo, Oslo, Norway; 3 Norwegian School of Theology, Oslo, Norway; 4 Division of Mental Health and Addiction, Oslo University Hospital, Oslo, Norway

## Abstract

**Background:**

Collaboration between general practice and mental health care has been recognised as necessary to provide good quality healthcare services to people with mental health problems. Several studies indicate that collaboration often is poor, with the result that patient' needs for coordinated services are not sufficiently met, and that resources are inefficiently used. An increasing number of mental health care workers should improve mental health services, but may complicate collaboration and coordination between mental health workers and other professionals in the treatment chain. The aim of this qualitative study is to investigate strengths and weaknesses in today's collaboration, and to suggest improvements in the interaction between General Practitioners (GPs) and specialised mental health service.

**Methods:**

This paper presents a qualitative focus group study with data drawn from six groups and eight group sessions with 28 health professionals (10 GPs, 12 nurses, and 6 physicians doing post-doctoral training in psychiatry), all working in the same region and assumed to make professional contact with each other.

**Results:**

GPs and mental health professionals shared each others expressions of strengths, weaknesses and suggestions for improvement in today's collaboration. Strengths in today's collaboration were related to common consultations between GPs and mental health professionals, and when GPs were able to receive advice about diagnostic treatment dilemmas. Weaknesses were related to the GPs' possibility to meet mental health professionals, and lack of mutual knowledge in mental health services. The results describe experiences and importance of interpersonal knowledge, mutual accessibility and familiarity with existing systems and resources. There is an agreement between GPs and mental health professionals that services will improve with shared knowledge about patients through systematic collaborative services, direct cell-phone lines to mental health professionals and allocated times for telephone consultation.

**Conclusions:**

GPs and mental health professionals experience collaboration as important. GPs are the gate-keepers to specialised health care, and lack of collaboration seems to create problems for GPs, mental health professionals, and for the patients. Suggestions for improvement included identification of situations that could increase mutual knowledge, and make it easier for GPs to reach the right mental health care professional when needed.

## Background

Problems associated with lack of collaboration among mental health professionals have been recognised for many years [1-3]. Although Norway is ranked among the highest of all OECD nations in public health spending per capita, many Norwegians do not receive adequate help and treatment [1].

Mental healthcare in Norway operates at two levels: primary health care and specialist health services. Primary health care includes the services provided by GPs and public and mental health nurses in the municipalities. The majority of GPs in Norway work in group practice (85 %) [4]. Specialist health services include outpatient clinics and psychiatric hospitals, and are organised in five regions with hospitals responsible for the delivery of services in designated catchment areas. In Norway, as in e.g. United Kingdom, France and Canada, GPs are often the first contact and play a central role in the management of psychiatric patients; they diagnose, initiate and continue medical treatment, are the gate-keepers to specialist care, and are supposed to know about available services offered in primary and secondary health care and to cooperate with outpatient clinics and acute wards, among other facilities [5,6].

In 1999 the Norwegian government initiated a restructuring and strengthening of the mental health services, the primary goal of which was to improve the quality of and access to these services. Even if the government did not make the GPs a part in this reform, the GPs play a main role as one of the primary health care service providers. The Evaluation of the reform shows major growth in the number of other professionals working at both levels of mental health care [7]. This increase leads to an expansion of services, and raises some challenges concerning collaboration and communication both within the two levels and between the primary and specialist level. An expansion of services requires more employees, which in turn can weaken their knowledge about each other and each other's specific service offers (mutual knowledge). Mutual knowledge involves knowing each other and information of each other's competence, systems, possibilities and restrictions.

As noted in The World Health Report, we should avoid a too excessive focus on specialised health services to maintain sustainable health services [8]. Municipal and GP programs aimed at reducing possible risk factors for mental health problems, and thereby preventing mental illness, may reduce the need for more expensive specialised services. According to the Norwegian Ministry of Health, better coordination between service levels is one of the most important areas to develop in the future [1].

Since the outpatient clinic often is the door to specialist mental health service in Norway [9], it is a crucial point for the GPs to succeed getting into contact with a competent person [10]. A study from France found that the GPs were in need for collaboration in 50 % of all their patients with mental health problems, indicating a great need for advice. The GPs were more willing to care for patients with mental health problems without sending referral if they received collaborative services from mental health professionals. The study concluded that there is a need for targeted collaboration between GPs and mental health professionals [6]. Even in Italy, where GPs do not have a gatekeeper function, they found that structured collaboration between GPs and mental health professionals had the potential to better meet the needs of people with mental health problems [5]. The collaboration consisted of consultation liaison service based on supplying diagnostic consultation and therapeutic interventions in support of GPs. In a study from the Netherlands, where different collaborative models were investigated, a model where trained mental health professionals had face-to-face contact with the GPs resulted in significantly higher satisfaction with services among general practitioners, shorter referral delay, reduced time in treatment, fewer appointments, and consequently lower treatment costs [11]. All these studies illustrate a collaborative practice where mental health professionals and GPs meet face to face and share their competence in the best interest of the patients.

In a recent study from Norway including 100 GPs interviewed by phone, the researchers asked only one question: "What can be done to improve treatment of mental disorders in primary health care?" The researchers concluded among other things, that there is a need for better co-operation between primary and secondary health care [12]. National surveys among Norwegian GPs evaluating outpatient clinics in mental health care, show that GPs in general are disappointed with waiting time, communication and cooperation [13]. Another survey shows that GP and patient ratings of health care services are highly correlated [14]. The aim of this qualitative study is to investigate strengths and weaknesses in today's collaboration, and to suggest improvements in the interaction between GPs and specialised mental health service.

## Methods

### A qualitative method

We chose a qualitative study because it can give more precise descriptions of content in collaborative challenges experienced by the GPs and mental health professionals. Focus groups are well suited for eliciting people's opinions and experiences, or for searching for deeper understanding of opinions and attitudes toward certain issues [15]. The strength of focus groups is the ability to capitalise on the interactions, discussions and relationships among group participants [16].

### Setting

The catchment area of Innlandet Hospital Trust, where this study was conducted, serves citizens in two counties with a total population of 378.000: Hedmark (192 000) and Oppland (186 000). The focus groups consisted of GPs and professional mental health workers in two municipalities strategically selected by the researchers to represent the population, infrastructure and location of Innlandet Hospital Trust. This also gave the study a broad range of experiences from a variety of professions and both levels of mental health services. One of the municipalities is rural with a population of 6.700, and the other municipality is a town with a population of 19.700. Specialist health services were represented by professional mental health workers at the acute wards and the outpatient clinic serving the population in the selected GP's. Each focus group session lasted 60 to 90 minutes, was audio recorded and transcribed for further analysis. The moderators asked two main questions:

1. What are the strengths and weaknesses in the collaboration between GPs and the specialist mental health care (outpatient clinics and acute wards) according to your experiences?

2. In what ways do you think this collaboration can be improved in order to enhance the quality of the mental health care services in your local situation?

### Description of the groups

Data were drawn from six focus groups engaged in a total of eight discussions (see table 1).

**Table 1:** Participants

**Group no**.	No. of participants (no. invited)	Location	Participants profession	Level of care
1	5 (6)	GPs rural	GPs	Primary
2	5 (6)	GPs town	GPs	Primary
3	6 (8)	Municipal psychiatric service	Specialist nurses in psychiatry	Primary
4	3 (4)	Outpatient clinic	Specialist nurses in psychiatry	Specialist
5	3 (3)	Acute ward	Specialist nurses in psychiatry	Specialist
6	6 (14)	Acute ward	Physicians on call	Specialist

The table shows group number, number of participants, number of participants invited (in brackets), location, participants' profession and level of care. Group number 1 and 2 consisted of a total of 5 different participants, and met twice with 4 participants in each focus group. Over all 28 different participants participated.

Three groups represented the primary level of care (see table 1). One group consisted of five GPs from the rural municipality, and the other consisted of five GPs from one well established group practice in the town municipality. The third group comprised of six professional mental health workers in mental health care at the primary level, with a mixed background from the same municipalities as the GPs. Three groups represented specialist health services, and comprised professional mental health workers. One group consisted of three nurses at an outpatient clinic, the second of three head nurses from each department in the acute ward, and the last of six resident physicians doing on call service in the acute ward. The focus groups comprising mental health professionals met once; the GPs' focus group met twice, under a multistage focus group process [17]. This was done to strengthen the focus on GPs, and giving them the opportunity to reflect on their first interview. Each group consisted of 3 to 6 participants, with a total of 28 persons participating.

### Leading the groups

The focus groups were led by a moderator (KK), whose primary role was to introduce the questions and to ensure that every focus group member was heard and that the participants' conversation were focused on the main purposes of the study. In this study, at least one co-researcher (TF) participated in the focus groups, asking additional questions, observing the atmosphere and interaction among participants, and writing brief notes. Rather than relying on nods or monosyllabic responses, the researchers asked follow-up questions to ensure that the information was understood. The researchers also conducted brief summaries after each main question, allowing participants to comment on the summary.

### Analysis

The focus group material was analysed using systematic text condensation [18-21]. Systematic text condensation is a process where a full transcribed text is read as a whole and condensed to meaningful units, through categorisation and coding. The authors worked together in this process, and discriminated units with meaning with a special focus on collaboration between GPs and mental health professionals. The units of meaning were sorted in categories to describe the groups statements in regard to the phenomena investigated.

The Norwegian Regional Committee for Medical Research Ethics approved this study, and every participant signed an informed consent form.

## Results

The participants shared their experiences about interaction with other professionals within mental health care. From the analysis three thematic clusters emerged. One cluster of experiences dealt with situations in which participants commented upon interpersonal knowledge and communication. Another cluster of experiences covered mutual accessibility, including ambulatory care. A last cluster of experiences covers unfamiliarity with existing system and resources.

### Interpersonal knowledge and communication

Our findings revealed that participants had occasionally experienced common consultations were they met the patient and a collaborative partner face to face. More interesting the participants reported positive experiences with these consultations. In situations where the participants knew the collaboration partners either by meeting them briefly or even only being familiar with their names, the communication tended to be easier, faster and more effective than in situations in which they had no knowledge of each other. One GP said:

*I think it's much easier to have a dialogue when I know who I'm talking to*.

The participants expressed benefits of meeting each other face to face and hearing about each other's possibilities, treatment plans and organisation. Recognising and sharing situations in which colleagues have experienced mutual knowledge as beneficial for communication and collaboration was an eye opener for GPs and mental health professionals. Despite this none of the participants mentioned any regularity in meetings or common consultations that included meetings with GPs. A participant from the outpatient clinic said:

*We have invited other professionals to information meetings, but again it's the old saying that GPs are so busy, so they are not invited*.

The participants from the outpatient clinic and primary mental health services had recently started a joint meeting series, which they found promising.

### Mutual accessibility (including ambulatory care)

Both GPs and psychiatric nurses at outpatient clinics experienced problems when they needed to contact physicians and specialists in the acute wards. The focus groups with GPs were dominated by outspoken frustration over unavailable mental health professionals, especially competent consultants. The GPs spoke about a general need for contact before referring a patient to clarify where to refer a patient or preparing the acute ward for an incoming patient. The GPs had also experienced situations where entering specialised service could be avoided through contact between GPs and mental health specialists, by providing GPs with advice for further treatment. Direct cell phone numbers for reaching mental health specialists were proposed to ease the situation. A GP said:

*Professionals in specialist mental health care should be trained to address themselves as consultants*.

GPs said that telephone calls were transferred from one person to another, and they often ended up with a secretary saying that the message would be delivered to the right person. Meanwhile, the patient had to wait for an answer while the GP handled other patients, and the GP could be occupied when the specialist phoned back. Neither GPs nor mental health professionals considered this procedure to be optimal. One GP said:

*I can't sit and wait for 15 minutes for a phone call about an acute situation. It's frustrating both for me and the patient. The patient is pending, in and out of the office, sometimes kept an eye on by the police. It's a messy situation*.

There were also problems the other way around as physicians on call in the acute ward expressed problems getting in contact with GPs. To establish contact was especially important in the process of discharging a patient, but due to problems getting in touch with GPs this was not prioritised. One physician on call said:

*It is not so easy to get in touch with GPs. You end up in line with everyone else*.

Both GPs and mental health professionals expressed the need for more ambulatory services, bringing specialised mental health services closer to the patients, GPs and other municipal services. A specific problem with long distances is that some patients find it difficult to travel to premises where specialised mental health treatment is offered. Distances and opportunities to travel were mentioned as factors that increased this problem. This problem was not mentioned by GPs working in the municipality where specialised mental health care was localised, however, reinforcing the impression that localisation and distance do matter. One GP said:

*It would be easier if you knew that, for example, every 14^th ^day a specialist in mental health care would come here to meet patients and talk to us*.

According to the participants, regular ambulatory services could also give service partners the opportunity to meet each other both in formal situations as an appointment and in informal settings as lunch breaks, and thereby increase mutual knowledge.

### Unfamiliarity with existing system and resources

Mental health professionals working in the acute ward expressed concerns with the system for in-house training. The head nurses in the departments had all experienced problems due to lack of training of physicians on call in the acute ward. One head nurse said:

*I have experienced standing outside the building, pointing at another building they are supposed to be at*.

Physician on call said that an updated telephone list with all available "secret" numbers to GPs existed, but several of them did not know of that list. This reinforces the impression that there are shortcomings in the system for in-house training of physicians on call in the acute psychiatric ward.

Participants revealed their lack of knowledge about possible co-operative partners and other mental health services in their region. Some of the GPs even lacked information about the location of the acute psychiatric ward, and were unfamiliar with the educational level of nurses working in specialised and municipal mental health care. Primary care nurses in the municipalities presumed that a large service system could cause some problems. One primary care nurse said:

*There is no lack of professionals to handle the patient, but the clue is to put things in a system where no one falls away*.

The participants from primary mental health services expressed a need for meeting places with GPs, and hypothesised that this also could give GPs information on services offered in their municipality. GPs on the other hand expressed a need for consultative services, especially from psychiatrists, because as one GP said:

*Almost every time medication is a topic*.

Participants in the focus groups also suggested routines and methods like direct cell-phone numbers, updated phone lists and allocated times for telephone consultation in order to reduce the time spent talking to what they regarded as irrelevant people, or waiting for someone to call back.

## Discussion

Face to face contact between GPs and mental health professionals seem to ease collaboration, and thereby better meet the needs of patients. Mutual problems with accessibility were experienced. There seems to be no systematic model for collaboration with regular meetings between GPs and mental health professionals, common consultations and regular ambulatory services. Participants also revealed being unfamiliar with existing systems like updated phone lists, possibilities for common consultations and possible collaborative partners.

Our findings suggest several weaknesses in interaction and communication between GPs and mental heath professionals, and can serve to support the national focus on improved coordination between primary and specialist levels of care. Opportunities to exchange views and knowledge with mental health professionals are an important tool for GPs, especially since they are gatekeepers to special health care. A result from this study indicates problems with phone calls between GPs and mental health professionals. Every time a GP fails in their attempt to reach mental health professionals by phone, the threshold could increase for new attempts.

There seems to be a potential in common consultations and regular meetings between primary and specialist levels of care. Common consultations among the GP, the professional in mental health care, and the patient, create knowledge and competence sharing between service partners and coordinates treatment for the patient. The benefit of meetings and contact could also make communication easier, create more positive attitudes and reduce the probability of misunderstandings and disagreements. Regular meetings can also reduce problems related to knowledge of other primary mental health services and professionals as a result of the increasing number of professionals in mental health care. Mental health professionals attached to a primary care practice and operate as part of the extended primary care team like in the Netherlands [11], or regular ambulatory service with opportunities of common consultation can give GPs in Norway the consultation and communication they need to enhance the quality of the mental health care services.

An excessive focus on specialised health services can serve to increase problems related to mental health services in the municipalities. GPs in cooperation with municipal services can reduce possible risk factors and prevent mental illness, treat mildly and moderate ill patients, and thereby reduce the need for specialised services. Providing GPs with collaborative mental health professional partners have the potential to further reduction in patient referrals to specialised mental health services [6]. The Norwegian eight year spending plan was initiated and started without GP involvement, and this may have led to collaboration problems between GPs, other municipal and specialised mental health services. Nearly all GPs in a Norwegian study suggested that there is a need for improvements in treatment of mental disorders in primary health care [12]. Of these; 57 % suggested improved co-operation between primary and secondary health care, and 40 % increased knowledge and competence among GPs. This is in line with the finding in our study, and therefore some strategies for improving collaboration and communication among GPs and mental health professionals are proposed. The models from the studies in Italy [5] and the Netherlands [11] should be given attention also in a Norwegian setting.

### Limitations and relevance

The limitations of our study lie primarily in the informants' willingness and motivation to reveal their feelings, beliefs and opinions to the researcher and the rest of the group. In this study, participants were informed that the focus group leaders were connected to specialised mental health care in the region. This may have increased the impression that the interviewers were in some way responsible for the situation, although the participants seemed to speak freely and were engaged. Because we wanted to highlight the cooperation between primary and specialist health workers, we did not include patients or patient organisations, as was done in an earlier study [10].

An aim of this study was to better describe strengths and weaknesses in today's collaboration. Even though there may be limitations in regard to generalisation of the results, the sample of participants were recruited from typical catchment areas, reflecting both rural and town societies. None of the results in our study contradicts findings in other studies, but can be valuable in a better description of the problems observed with collaboration between primary care and mental health professionals. Our findings are in line with other studies which can argue for reliability in our study. Furthermore our findings can have transferability and importance for further research in the field.

## Conclusions

The study shows how coordination is experienced by GPs and other mental health professionals involved, and exemplify what can happen when communication is far from optimal. Before GPs initiate and begin treatment of mental health problems, they are sometimes in need of advice and consultation, which should be provided by mental health specialists. Problems with accessibility may reduce the frequency of asking for help when needed. On the other hand, an increase in accessibility at the specialist level of mental health services may lead to more consultative services by telephone and less direct patient contact for mental health specialists. This may be a temporary problem, because GPs competence will be strengthened if they can obtain better consultative services. The important issue is to provide GPs with help when they need it, resulting in better services to patients when they need it. This study pinpoints arguments for better coordination between GPs and mental health care professionals. Creating mutual knowledge through regular and systematic collaboration could solve many of the communication problems that were found in this study.

## Competing interests

The authors declare that they have no competing instructions.

## Authors' contributions

TF participated in interviews, transcribed and analyzed them. He also wrote the paper. LD participated in some interviews and analyzed all interviews. ORH analyzed interviews. KK conducted the interviews and analyzed them. LL participated in some interviews and supervised the overall project. All authors have read and approved the final manuscript.
